# The Editor’s Choice for Issue 3, Volume 7

**DOI:** 10.3390/ijns7040084

**Published:** 2021-12-20

**Authors:** Ralph Fingerhut

**Affiliations:** SYNLAB MVZ Weiden, Zur Kesselschmiede 4, 92637 Weiden, Germany; Ralph.Fingerhut@synlab.com

Dear Readers: Choosing one paper from a total of 28 papers published in the third issue of Volume 7 was quite a challenge. The papers in the third issue cover a lot of different topics, which all are relevant for the advancement of newborn screening (NBS), and as my co-editor Can Ficicioglu has already mentioned for the second issue [1], the quality of papers published in the third issue has remained at a high level, which can be estimated by the number of reads/downloads. The papers in the third issue already have more than 22,000 reads, and even the most recent article from September already has 564 reads in less than 3 months. However, from all the interesting topics that included single papers on methodology, lysosomal storage diseases, peroxisomal disorders, long-term follow-up, congenital hypothyroidism, methylmalonic acidemia, homocystinuria, and a bigger number of papers on NBS for spinal muscular atrophy (SMA) and severe combined immunodeficiency (SCID), I want to highlight the paper from Allan Meldgaard Lund et al. [2] which describes the use of molecular genetic analyses in the Danish routine NBS. This paper describes not only the introduction of molecular genetic testing as a second-tier method into NBS, which has already been described for other countries [3,4,5,6,7], but it also highlights the challenges and possible pitfalls of second-tier genetic testing. In addition, the authors also discuss the possibility of reversing this approach into first-tier genetic testing and second-tier metabolite testing. 

The reduction in false-positive results is one of the main advances of second-tier testing, although this is not restricted to second-tier genetic testing, but has been shown for various other second-tier methods [8,9]. In addition, the authors also address all possible pitfalls and drawbacks of genetic testing, like carrier detection, detection variants of unknown significance, and incidental findings. 

I would like to highlight two additional topics: One is the possibility to include treatable diseases into NBS with first-tier genetic screening, when there is no known biochemical marker [10,11,12,13]. This is partly already discussed by the authors, and partly already implemented into NBS programs, with SCID and SMA screening. The second point, which is, if at all, only discussed very rarely, is the problem of shortage of blood, with the steadily increasing number of target diseases. First-tier genetic NBS has the potential to solve this problem. Instead of 4-5 spots on the blood collection device (formerly called “Guthrie card” or “filtercard”), then 1-2 spots would be absolutely sufficient [14]. 
